# Impact of deoxynivalenol in a calcium depletion and repletion nutritional strategy in piglets

**DOI:** 10.1093/jas/skae099

**Published:** 2024-04-13

**Authors:** Béatrice Sauvé, Frédéric Guay, Marie-Pierre Létourneau Montminy

**Affiliations:** Department of Animal Sciences, Université Laval, Québec (QC), CanadaG1V 0A6; Department of Animal Sciences, Université Laval, Québec (QC), CanadaG1V 0A6; Department of Animal Sciences, Université Laval, Québec (QC), CanadaG1V 0A6

**Keywords:** bone mineralization, calcium, deoxynivalenol, depletion, phosphorus, piglets, repletion

## Abstract

This study evaluated the effect of dietary calcium (**Ca**) levels and deoxynivalenol (**DON**) contamination on Ca and phosphorus (**P**) utilization and bone mineralization in piglets. During an initial 13-d depletion phase, 64 piglets (15.7 ± 0.7 kg) received a control (DON−) or DON-contaminated treatment (DON+, 2.7 mg DON/kg) with either a low Ca (Ca−, 0.39%) or normal Ca level (Ca+, 0.65%) with a constant digestible P level (0.40%). A second group of 16 piglets received DON− or DON+ treatments for 9 d for gene expression analysis. During the subsequent 14-d repletion phase, all piglets were fed a Ca+ DON− diet containing 0.65% Ca and 0.35% digestible P without DON. After 5 d of the depletion phase, the absorption of P (DON × Ca; *P* < 0.05) and Ca was increased by the Ca− (*P* < 0.01) and DON+ (*P* < 0.01) diet. After 13 d, feed conversion ratio (*P* < 0.01) and average daily feed intake (*P* = 0.06) tended to decrease with the Ca− diet. The bone mineral content (**BMC**) gain was decreased by Ca, especially with Ca− DON + (DON × Ca, *P* < 0.05). The P absorption was increased by Ca− DON + (DON × Ca, *P* < 0.01), although the P retention efficiency was only increased by Ca+ DON + (DON × Ca, *P* < 0.001). The absorption of Ca was increased by DON+ (*P *< 0.001), and the Ca efficiency was increased by Ca− DON− (DON × Ca, *P* < 0.01). After 9 d, the gene expression of intestinal claudin 12 (*P* < 0.01) and CYP24A1 (*P* < 0.05), femur cortical RANKL (*P* < 0.05) and OPG (*P* = 0.06), and renal calbindin D9K (*P* < 0.05) and Klotho (*P* = 0.07) were decreased by DON+. The Ca (*P* = 0.06) and magnesium (*P* < 0.01) concentrations were decreased by DON+, and the Ca (*P* = 0.06) and P digestibility (*P* < 0.01) were increased. After the repletion phase, Ca− piglets recovered their BMC deficit, but not those receiving DON+ (DON × Ca; P = 0.06). The Ca (*P* < 0.05) and P (*P* = 0.06) retention efficiency tended to increase with Ca−. The absorption of Ca and P was increased by Ca− and DON+ (DON × Ca, *P* < 0.05). The results show that piglets increased their Ca and P utilization efficiency, allowing them to recover the BMC deficit caused by Ca−, but not when the piglets were exposed to DON. Pigs previously receiving Ca-deficient diet with DON still have lower body Ca and P, leading to elevated calcitriol concentrations and enhanced Ca and P intestinal absorption. The fact that DON decreased the expression of genes implicated in Ca intestinal and renal transport and P excretion after 9 d can potentially explain the reduced plasma Ca concentration.

## Teaser

A short calcium (**Ca**) depletion phase can improve the utilization efficiency of Ca and phosphorus (**P**) in piglets by increasing the retention of these nutrients throughout both the Ca-depletion period and the subsequent recovery phase, which allows the recovery of bone mineralization. However, a diet contaminated with deoxynivalenol (**DON**) prevented pigs from recovering from their bone mineralization deficit during the Ca-depletion phase, even when Ca and P digestibility increased, as demonstrated by reduced expression of genes involved in Ca intestinal absorption, renal transport, osteoclastogenesis, and P excretion.

## Introduction

In the past decade, the occurrence of mycotoxins has increased following climate changes in North America (Gruber-Dorninger et al., 2019; Zingales et al., 2022), making the use of by-products and low-graded grains more prone to introduce mycotoxins in animal feed. The variations in humidity levels and warmer climates associated with climate change have favored the growth of certain fungi, including the *Fusarium* fungus (Zingales et al., 2022). The mycotoxin deoxynivalenol (DON) produced by the *Fusarium* fungus is resistant to high temperatures, various pH levels, and processing; therefore, it is widely present in animal feeds (Pestka, 2007; Sobrova et al., 2010). This secondary metabolite is part of the trichothecene group and is commonly found in wheat, barley, oats, and corn (Mishra et al., 2014), but at concentrations that usually do not exceed 1.0 mg/kg (Zhao et al., 2021), which is the maximum limit recommended by the Canadian Food Inspection Agency in finished product for pigs, while 5.0 mg/kg is recommended in grains and by-products with a 20% inclusion in the pigs’ diet (Pasternak et al., 2018; Ministry of Agriculture, Food and Rural Affairs of Ontario, 2022). In pigs, DON contamination is known to decrease feed intake, leading to reduced growth (Le Thanh et al., 2015; Serviento et al., 2018), even at doses as low as 1.0 mg/kg of feed (Accensi et al., 2006). In addition to these known effects, Le Thanh et al. (2015) observed that DON contamination (4.0 mg/kg) improved Ca retention and reduced Ca and P excretion in piglets. Our previous study also showed that piglets fed with DON-contaminated feed (4.9 mg/kg) had decreased gene expression related to Ca and P intestinal and renal absorption, and increased bone mineralization relative to body weight, suggesting that DON impacts phosphocalcic metabolism (Sauvé et al., 2023).

Regulation of Ca and P metabolism involves various hormones, the key ones being parathormone (**PTH**), calcitonin, and the active form of vitamin D (calcitriol; 1,25-OH_2_-D_3_) (Karsdal et al., 2008; Fukumoto, 2014; González-Vega and Stein, 2014). Plasma Ca variations are detected by the Ca-sensing receptor (**CaSR**) in the parathyroid glands. During hypocalcemia, CaSR is inactivated, which stimulates PTH secretion and subsequently the synthesis of calcitriol in the kidneys by the enzyme 1α-hydroxylase, regulated by the CYP27B1 gene (Christakos et al., 2010; Pu et al., 2016). An increase in PTH and calcitriol stimulates Ca renal reabsorption and Ca intestinal absorption (González-Vega and Stein, 2014; Pu et al., 2016). As a negative feedback mechanism, calcitriol then decreases PTH synthesis (Nussey and Whitehead, 2001). PTH and calcitriol also stimulate bone resorption to release Ca and P into the bloodstream by increasing the activity of osteoclasts and inhibiting osteoblasts (González-Vega and Stein, 2014; Pu et al., 2016). Osteoblasts and osteoclasts are responsible for the production and resorption of bone, respectively, in the bone remodeling process. Osteoclast differentiation is initiated by the receptor activator of the NFκB ligand (**RANKL**) through its interaction with the RANK receptor (O’Brien, 2010). When plasma Ca concentration is elevated, osteoclast activity is inhibited and osteoblast activity is stimulated by calcitonin secretion (González-Vega and Stein, 2014). Also, hyperphosphatemia and increased PTH and calcitriol activity increase the production of fibroblast growth factor 23 (**FGF23**) from osteocystic cells in bone, which is responsible for the decrease in P renal reabsorption (Haussler et al., 2012; Fukumoto, 2014). The FGF23 hormone then decreases calcitriol biosynthesis by inhibiting CYP27B1 and inducing the 24-hydroxylase enzyme regulated by CYP24A1 gene in kidney tissues (Haussler et al., 2012).

Pigs can enhance their digestive and metabolic utilization during a nutritional deficiency (Patterson et al., 2008). Therefore, a reduction in Ca or P levels for a specific period of time will lead to the depletion of bone reserves, which is called a “depletion phase,” and will induce regulations resulting in increased utilization efficiency of Ca and P, by increasing their intestinal absorption, their renal reabsorption and the bone resorption process. This depletion phase is followed by a recovery phase, which is called the “repletion phase,” where Ca and P are provided at or over recommended dietary levels. By reducing Ca intake below recommended levels while maintaining an adequate Ca:P ratio, performance should not be impacted during the depletion phase (Lagos et al., 2019) but bone mineralization should be decreased. During the repletion phase, pigs should recover from their bone mineralization deficit, as has previously been observed in growing pigs fed a Ca- and P-deficient diet, depending on the duration of the repletion phase (Létourneau-Montminy et al., 2011; Létourneau-Montminy et al., 2014;Gonzalo et al., 2018).

As previous studies from our team observed effect of DON contamination on Ca and P metabolism, using different Ca levels in DON-contaminated feed could modify the pigs use of Ca and P by modifications in bone mineralization process and intestinal and renal absorption. The objective of this study was to investigate the effects of a depletion–repletion protocol in the presence of dietary DON contamination on growth performance, Ca and P digestibility and retention efficiency, bone mineral content (**BMC**), and blood parameters. A different group of pigs was used to evaluate the effect of dietary DON on gene expression related to intestinal and renal absorption of Ca and P and to bone remodeling to understand the underlying mechanisms.

## Material and Methods

### Experimental diets

The experiment was performed at the Centre de Recherche en Sciences Animales de Deschambault (Quebec, Canada) and followed the guidelines of the Canadian Council on Animal Care (2009); the protocol was approved by the Institutional Animal Care and Use Committee at Laval University (protocol #2020-648). All Ca+ diets fulfilled the NRC requirements (NRC 2012; Table 1), while Ca diets were 27% lower than the NRC requirements for Ca. Eighty mixed gender piglets ([Yorkshire × Landrace] × Duroc; Olymel St-Hyacinthe, Qc, Canada) weaned at 21 d of age were distributed in 40 pens (2 piglets/pen) and fed with a commercial diet (Agri-Marché, St-Isidore, QC, Canada) for 3 wk. One week before trial, piglets were distributed depending on their weight into 4 complete blocks with 8 repetitions for experiment 1, and into 2 separate complete blocks with 4 repetitions for experiment 2 according to a randomization method (Kim and Lindemann, 2007). In experiment 1, 64 of these piglets (15.7 ± 0.69 kg, 42 d of age) received one of the following four treatments in a 2 × 2 factorial design during an initial 13-d depletion phase: control (DON−) or DON-contaminated treatment (DON+; 2.72 mg/kg from naturally contaminated wheat; aflatoxins < 1.0 ppb, zearalenone 0.03 ppm, fuminosine 0.1 ppm, ochratoxin 0.01 ppm, HT-2 < 0.06, T-2 < 0.06, diacetoxyscirpenol < 0.06 ppm, sterigmatocystin < 0.03 ppm, mycophenolic acid < 0.03 ppm) with either a low Ca (Ca−, 0.39%) or normal Ca level (Ca+, 0.65%), and a constant digestible P level (0.40%). During a second 14-d repletion phase, all piglets were fed a similar diet containing 0.65% Ca and 0.35% P without DON contamination. In experiment 2, 16 piglets (15.6 ± 0.31 kg) received DON− or DON+ treatments (same diet as experiment 1 with naturally contaminated wheat) for 9 d with normal Ca level (0.65%) to collect blood samples; these pigs were euthanized afterwards to collect cortical femur, jejunum, liver, and kidney tissues to analyze the gene expression.

**Table 1.** Composition of experimental diets

	Ca+ DON−	Ca+ DON+	Ca− DON−	Ca− DON+	Ca+ DON−
Ingredient (kg)	Phase 1				Phase 2
Corn	385.85	374.10	397.70	386.15	541.65
Soft wheat	300.00		300.00		150.00
Soft wheat contaminated		300.00		300.00	
Soybean meal	217.20	223.70	216.40	222.85	211.90
Insoluble ash (celite)	30.00	30.00	30.00	30.00	30.00
Fat (vegetable oil)	24.70	29.70	20.60	25.60	25.75
Limestone	6.90	6.90			8.00
BioPhos (Monocalcium phosphate)	15.85	15.85	15.85	15.80	13.85
Salt	6.20	6.20	6.20	6.20	6.25
Lysine-HCl	5.50	5.50	5.50	5.50	5.10
dl-Methionine	1.40	1.50	1.40	1.45	1.30
Threonine	2.15	2.15	2.10	2.10	2.00
l-Tryptophane	0.45	0.50	0.45	0.50	0.55
l-Valine	0.80	0.90	0.80	0.85	0.75
Choline chloride 60%	0.50	0.50	0.50	0.50	0.40
Vitamin–mineral premix^1^^,^^2^	2.50	2.50	2.50	2.50	2.50
Calculated composition (%)					
Total calcium	0.65	0.65	0.39	0.39	0.65
Calcium with phytase	0.65	0.65	0.39	0.39	0.65
Total phosphorus	0.68	0.68	0.68	0.68	0.63
Digestible phosphorus	0.40	0.40	0.40	0.40	0.35
Sodium	0.25	0.25	0.25	0.25	0.25
Analyzed composition					
Calcium (%)	0.72	0.70	0.42	0.37	0.62
Phosphorus (%)	0.70	0.68	0.71	0.61	0.63
Deoxynivalenol (mg/kg)	0.22	2.92	0.19	2.52	0.63

^1^Provided per kilogram of diet: vitamin A 8,500 UI; vitamin D 1,500 IU; vitamin E 63.8 UI; vitamin K menadione 4.0 mg; riboflavin B2 7.5 mg; pyridoxine B6 4.0 mg; folic acid 1.25 mg; niacin B3 40.0 mg; thiamin B1 2.5 mg; biotin 200.0 mg; pantothenic acid 25.0 mg.

^2^Provided per kilogram of diet: calcium 0.03%; zinc 250.1 mg; iron 155.5 mg; copper 124.4 mg; manganese 45.3 mg; selenium 0.3 mg; cobalt 0.2 mg; iodine 0.5 mg.

### Animal management

Feed was manually distributed and was noted daily. Feed refusal was evaluated, and piglets were weighed at the beginning and the end of each phase. Blood samples were taken from one piglet per pen (32 units) by jugular venipuncture (BD Canada, Mississauga, ON, Canada) before the BMC of that same piglet per pen was evaluated with dual-energy X-ray absorptiometry (DXA; Discovery W, Hologic, Massachusetts) at the beginning and end of each phase, which is precise for BMC evaluation (Kasper et al., 2021). The scanned pigs received an injection of azaperone after the blood sample was taken (2.2 mg/kg bodyweight; Stresnil; Jansen-Cilag, Neuss, Germany) depending on their weight to calm them at least 10 min before they were anesthetized by mask inhalation of sevoflurane (Sevorane; Abbott Laboratories, North Chicago, IL, USA) at an oxygen concentration of 7%. Once the pigs were unconscious, sevoflurane was replaced by isoflurane (IsoFlo; Abbott Laboratories) at an oxygen concentration of 5% to keep the pigs anesthetized during scans. The animals were scanned in prone position with the front legs along the sides of the body using whole-body mode. The difference of BMC between the end and beginning of each phase divided by the number of days of each phase represented the daily BMC gain per pig. In experiment 2, body weight and feed refusal were evaluated at the beginning and after 9 d of trial. Blood samples from the 16 piglets for plasma and serum were taken before euthanasia after 9 d by jugular venepuncture (BD Canada) with the same method mentioned above. The 16 piglets were then euthanized using a nonpenetrating captive bolt stunner. Tissue samples of liver, jejunum mucosa, kidney, and cortical and trabecular femur were taken to assess gene expressions related to P, Ca, and vitamin D metabolism. More specifically, liver tissues were taken on the right lobe, kidney tissues of the left one were taken in the cortex underneath the fibrous capsule, jejunum tissues were cut in the middle of the segment to have two 1.5 cm pieces and all tissues were cut into tiny pieces to facilitate RNA extraction. The femur tissues from tubular and cortical sections were cut into slices after being cleaned of muscle and cartilaginous cap for trabecular femur. Tissue samples were snap-frozen in liquid nitrogen directly after collection and put at −80 °C upon laboratory analysis.

### Digestibility and body composition analysis

An indigestible marker, acid insoluble ash (celite 3%, Probiotech International Inc., Saint-Hyacinthe, Qc, Canada), was incorporated into the experimental diet and grab samples of fresh feces were taken on days 5, 12, and 26 of the trial from 32 pens used for BMC and blood sampling in experiment 1. The apparent total tract digestibility (**ATTD**) of Ca and P was calculated using the following formula:


$$\begin{array}{l} Apparentdigestibility(\%)=\left[1-\frac{Nutrients_{feces}\times AIA_{feed}}{Nutrients_{feed}\times AIA_{feces}}\right]\times 100 \end{array}$$

The daily digestible P and Ca (intake, absorbed, and feces) was calculated for each day (days 5, 12, and 26). The Ca and P intake was estimated using average feed intake during each period and dietary mineral concentrations. The ATTD of Ca and P were used to estimate minerals absorbed and difference between daily mineral intake and absorbed was considered as daily mineral excreted in feces. Body lean and fat contents obtained from the DXA scan were used to determine whole-body protein (protein = 0.216 × lean) and lipid (lipid = 1.009 × fat) contents (Pomar and Rivest, 1996), and body mineral content was used to calculate the body Ca and P based on the model from Lautrou et al. (2020). The retention efficiency (%) of Ca and P was calculated by dividing the daily body gains of P and Ca by the daily feed intake of P and Ca estimated for each phase.

### Laboratory analysis

The collected feces were frozen at −20 °C before analyses and lyophilized for 7 d. Feces (0.50 g) were solubilized in HCl 4 N for 30 min and incinerated at 600 °C for 6 h (McCarthy et al., 1974). The Ca and P in the feed and feces were evaluated using inductive coupled plasma–optical emission spectrometer (**ICP-OES**) in a commercial laboratory (Activation Laboratories, Lancaster, ON, Canada). Plasma EDTA and serum tubes were centrifuged at 2,000 × *g* at 4 °C for 15 min. Plasma EDTA and serum samples were collected and kept frozen at −20 °C until assayed. Serum concentrations of DON and deepoxy-deoxynivalenol (**DOM-1**) were evaluated (Le Thanh et al., 2015). Phosphate, total calcium, and magnesium plasma concentrations were evaluated using ICP-OES. For this evaluation, 1 mL of plasma was used, and 0.5 mL 3N HCl, 0.5 mL 40% TCA, and 3 mL milliQ water were added and mixed between each addition. Samples were centrifuged at 7,500 × *g* for 20 min and analyzed by ICP-OES.

The serum samples of piglets were used to measure 25-OH-D_3_, called calcidiol, by high-performance liquid chromatography (**HPLC**, Agilent Technologies Canada Inc., Mississauga, ON, Canada) according to a method adapted from Horst et al. (1981). Plasma (500 µL) was mixed with 200 µL ethanol (100%) and 400 µL isopropanol. The solution was extracted with 800 µL hexane and evaporated using nitrogen. Then, 500 µL methanol was added to the dry tubes and evaporated. Finally, 125 µL 65% acetonitrile solution was added to each tube. Each sample was injected (75 µL) into an HPLC Agilent 1200 (Agilent Technologies, Santa Clara, CA, USA) with a mobile phase of acetonitrile and a gradient of 65% to 87% and then to 100% at a flow of 1.2 mL/min. Detection of 25-OH-D_3_ was performed at 264 nm. Concentrations of 1,25-(OH)_2_-D_3_ were evaluated in serum by the sandwich ELISA method (BioVendor, Brno, Czech Republic). Calcitriol extraction was done using 500 µL serum with the addition of 2 mL of a mix of di-isopropyl ether, cyclohexane, and ethyl acetate (50/40/10 volume/volume). The supernatant passing through the extraction column was washed with the same mix. Dichloromethane (500 µL) was added to the extraction column, then 500 µL milliQ water. Finally, 300 µL methanol was added to elute calcitriol. The resulting sample was used for the ELISA procedure.

### Gene expression analysis

The mRNA gene expression was quantified according to the method of Lessard et al. (Lessard et al., 2015). The tissue samples (50 mg), except for femur tissues, were homogenized in 1 mL TRIzol© (Thermo Fisher Scientific Inc., Carlsbad, CA, USA), 200 μL chloroform was added, and samples were centrifuged for 15 min at 12,000 × *g* at 4 °C. The aqueous phase was transferred into a new tube and 500 μL isopropanol was added. After centrifugation for 10 min at 12,000 × *g* at 4 °C, isopropanol was removed from the tubes and 75% ethanol was added for a 5-min centrifugation at 7,500 × *g* at 4 °C. DNase free water (50 µL) was added to dilute the pellet. Cortical and trabecular (including growth plates) femur tissues were homogenized using a Cryomill (Retsch, Haan, Germany). The samples were placed into the biggest jar (50 mL) with three 15 mm beads for grinding one at a time. The jar with the sample was cooled for 2 min at 5 Hz frequency, followed by three cryo-cycles of 1 min at 25 Hz and 30 s at 5 Hz. Homogenized femur samples (50 mg) were extracted with 1 mL TRIzol with the method mentioned above (Thermo Fisher Scientific Inc.). Reverse transcription was performed with the qScript Flex (Qiagen Beverly Inc., Cummings, MA, USA) with a 1 ng/μL mRNA concentration. The qPCR was performed with 10 μL PerfeCTa SYBR Green FastMix (Quanta Bioscience Inc., Gaithersburg, MD, USA), 1 μL cDNA, 1 μL designed primers (Table A1), and 8 μL RNAse-free water using the Lightcycler 480. The PCR cycling conditions were 10 min at 95 °C, followed by 50 cycles of 10 s at 57 °C or 58 °C for primer annealing (depending on the gene) and 20 s at 72 °C for primer extension; a melting curve step was added at 72 °C for 10 s and 94 °C for 5 cycles. A relative standard curve was established by serial dilutions of a cDNA pool and used to determine the mRNA expression levels.

Vitamin D receptor (VDR) gene expression was evaluated in all tissues. In the liver, only CYP2R1 (25-hydroxylase, activates vitamin D_3_ into 25-OH-D_3_ in liver) gene expression was evaluated, because the liver does not participate in the most important regulations of Ca and P absorption. In the kidney and jejunum mucosa, Klotho (FGF23 co-receptor), CALB-1 (Calbindin-1, Ca kidney transportation), S100G (Calbindin D9K, Ca transportation), and CYP24A1 (24-hydroxylase, degradation of calcitriol) gene expressions were assessed. The CYP27B1 (1α-hydroxylase, hydroxylation of 25-OH-D_3_ into 1-25-(OH)_2_-D_3_), SLC34A3 (Na-Pi type IIc, renal phosphate co-transporter), SLC8A1 (Na^2+^/Ca^2+^ 1 exchanger, transfers Ca to blood circulation), and TRPV5 (transient receptor potential vanilloid 5, entry channel of Ca into kidney) gene expressions were evaluated in the kidney. The SLC20A2 (Na-Pi type III transporter, intestinal and kidney absorption of P), CLDN2 and CLDN12 (Claudine 2 and 12, paracellular Ca absorption), and TRPV6 (transient receptor potential vanilloid 6, entry channel of Ca into the brush border) gene expressions were evaluated in the jejunum. The Klotho, CYP27B1, OTC (osteocalcin, osteoblastic bone formation), RANKL (receptor activator of nuclear factor-κB ligand, differentiation of osteoclasts), OPG (osteoprotegerin, antagonist to RANK receptor), and Runx2 (inhibits osteoblast maturation) gene expressions were evaluated in the trabecular and cortical femur tissues. Gene expressions were normalized with three housekeeping genes, GAPDH, β-Actin, and HPRT. The uniformity of the expression of housekeeping genes was tested with the geNorm analysis, part of Biogazelle’s qbase + 2.6 software (Biogazelle, Zwijnaarde, Belgium). The threshold for CV and M (geNorm expression stability value) was set to 0.2 and 0.5, respectively.

### Statistical analyses

In experiment 1, growth performance including average daily feed intake (**ADFI**), average daily gain (**ADG**), feed conversion ratio (**FCR**), and ATTD and retention efficiency (%) of Ca and P were analyzed in a factorial design ANOVA 2 × 2 (DON contamination and Ca levels as fixed effects), using a pen of 2 piglets as the experimental unit and blocks based on the initial weight of the piglets as a random effect with the MIXED procedure on SAS (SAS studio 2021, SAS Inst. Inc. Cary, NC), to assess the effects of DON, Ca, and their interactions. Plasma and serum concentrations, BMC, body Ca and P, and their gain (g/d) were also analyzed, using similar statistical analyses, in a factorial design 2 × 2 (DON contamination × Ca levels as fixed effects) for one piglet per pen as an experimental unit. In experiment 2, the experimental unit was the piglet with DON contamination as the fixed effect. A *P*-value less than 0.05 indicated a significant difference, whereas a *P*-value between 0.05 and 0.10 indicated a statistical trend. The hypothesis necessary for the application of analysis of variance (normality of residuals and homogeneity of variances) was verified in all cases. A Poisson distribution was used when necessary, with the Glimmix procedure on SAS (SAS studio 2021). Only one pen was removed from the study, as one of the two piglets in the pen died early in the study.

## Results

### Depletion phase

#### Growth performance and bone mineralization

During the depletion phase, no changes were observed for ADG, ADFI, and final body weight. and ADFI tended to decrease in the Ca− group (*P* = 0.056; Table 2). FCR was decreased in Ca− (*P* < 0.01) with a tendency for a higher reduction in the DON− group (DON × Ca; *P* = 0.069). The body protein content tended to be increased in the Ca− DON− group, while it was reduced in Ca− DON+ (DON × Ca; *P* = 0.074). The BMC was decreased in Ca− more drastically in DON+ (−20%) than in DON− (−6.3%; DON × Ca, *P* < 0.001). The BMC/kg of bodyweight (**BW**) was reduced in piglets in the Ca− group (*P* < 0.001). The body Ca and P contents were reduced in piglets in the Ca− group, especially with DON+ (DON × Ca, *P* < 0.01). Throughout the depletion phase, an interaction between DON and Ca showed that decreasing dietary Ca reduced BMC, body Ca, and body P gains (*P* < 0.01), especially with the DON+ diet (DON × Ca, *P* < 0.05). The Ca efficiency (%) in piglets receiving Ca− increased more in DON− (+ 67%) than in DON+ (+3.0%; DON × Ca; *P* < 0.01). The P efficiency (%) decreased in the Ca− DON+ group while it was increased in the Ca− DON− group (DON × Ca; *P* < 0.001).

**Table 2.** Impact of a Ca-deficient diet and deoxynivalenol (DON) contamination on growth performances and bone mineralization after a 13-d depletion phase^1^

Treatments	Ca+		Ca−		SEM	*P* value	Ca	DON × Ca
	DON−	DON+	DON−	DON+		DON		
Initial conditions								
Body weight (kg)	13.8	14.0	14.1	14.2	0.63	0.640	0.364	0.882
Body protein (g)	2479	2459	2494	2541	117.8	0.817	0.403	0.565
Body lipid (g)	2203	2450	2400	2276	122.3	0.440	0.898	<0.050
BMC (g)	221	242	222	224	7.7	0.148	0.279	0.223
BMC/BW (%)	1.60	1.74	1.57	1.58	0.06	0.205	0.112	0.262
Body Ca (g)	80.7	88.3	81.1	81.8	3.53	0.148	0.284	0.225
Body P (g)	66.6	70.3	67.1	67.9	2.73	0.199	0.556	0.394
Final conditions								
Body weight (kg)	22.4	24.0	24.1	23.4	0.95	0.500	0.412	0.088
Body protein (g)	4025	4361	4390	4262	174	0.407	0.292	0.074
Body lipid (g)	3438	3488	3484	3419	217	0.969	0.951	0.765
BMC (g)	357	382	334	305	7.89	0.749	<0.001	<0.001
BMC/BW (%)	1.61	1.60	1.39	1.31	0.04	0.222	<0.001	0.274
Body Ca (g)	130	140	124	112	3.47	0.764	<0.001	<0.001
Body P (g)	108	116	108	101	3.26	0.768	<0.01	<0.01
Overall								
ADG (g/d)	707	719	727	657	20.1	0.264	0.418	0.123
ADFI (g/d)	1187	1156	1127	1031	44.5	0.179	0.056	0.478
FCR	1.69	1.60	1.55	1.57	0.03	0.267	0.007	0.069
BMC gain (g/d)	9.99	10.5	8.39	6.02	0.41	0.130	<0.001	0.019
Body Ca gain (g/d)	3.65	3.86	3.06	2.23	0.22	0.153	<0.001	0.021
Body P gain (g/d)	3.02	3.41	3.00	2.45	0.14	0.593	<0.01	<0.01
Ca efficiency^2^ (%)	36.1	43.3	60.4	44.6	3.84	0.230	<0.01	<0.01
P efficiency^2^ (%)	29.8	39.5	33.3	29.4	1.69	0.052	0.030	<0.001

^1^Values are least square means.

^2^Efficency was calculated by ratio between mineral retained estimated by DXA on mineral intake.

ADFI: average daily feed intake; ADG: average daily gain; FCR: feed conversion ratio; BW: body weight; BMC: whole-body bone mineral content; SEM: standard-error means.

### Digestibility and blood parameters after 5 and 13 d

After 5 d of depletion, the daily P intake was reduced by DON+ only in Ca− (DON × Ca; *P* < 0.05; Table 3) and the fecal P was decreased by DON+ regardless of Ca levels (*P* < 0.001). The quantity of P absorbed was increased with DON+ only in Ca+ (DON × Ca; *P* < 0.05). The ATTD of P was increased in piglets receiving the Ca− with DON− (DON × Ca; *P* < 0.05). The daily Ca intake and fecal Ca were decreased by both DON+ (*P* < 0.01) and Ca− (*P* < 0.01). The quantity of Ca absorbed was also decreased by both DON+ (*P* < 0.01) and Ca− (*P* < 0.05). The ATTD of Ca was increased in piglets fed DON+ (*P* < 0.01) and Ca− (*P* < 0.05).

**Table 3.** Digestible phosphorus and calcium in pigs measured after 5 and 13 d of the depletion phase^1^

Treatments	Ca+		Ca−		SEM	*P* value	Ca	DON × Ca
	DON−	DON+	DON−	DON+		DON		
5 d								
Phosphorus								
Intake (g/d)	8.00	8.12	8.98	7.46	0.34	0.063	0.660	<0.05
Feces (g/d)	3.96	2.59	3.59	2.42	0.18	<0.001	0.185	0.602
Absorbed (g/d)	4.24	5.65	5.39	5.04	0.26	0.140	0.436	<0.05
ATTD of P (%)	50.5	68.2	60.3	67.4	1.65	<0.001	<0.05	<0.05
Calcium								
Intake (g/d)	10.6	7.41	7.59	4.63	0.46	<0.001	<0.001	0.857
Feces (g/d)	4.13	2.11	2.14	1.09	0.25	<0.001	<0.001	0.091
Absorbed (g/d)	6.33	5.36	5.43	3.53	0.38	<0.01	<0.05	0.360
ATTD of Ca (%)	59.4	71.7	72.2	76.2	2.6	<0.01	<0.01	0.152
13 d								
Phosphorus								
Intake (g/d)	10.2	8.76	9.12	8.39	0.39	<0.01	0.068	0.378
Feces (g/d)	4.26	3.26	3.89	2.61	0.21	<0.001	<0.05	0.489
Absorbed (g/d)	5.90	5.51	5.23	5.77	0.21	0.766	0.400	0.059
ATTD of P (%)	58.3	63.0	57.7	68.8	0.93	<0.001	<0.05	<0.01
Calcium								
Intake (g/d)	10.2	9.08	5.14	4.98	0.33	0.084	<0.001	0.183
Feces (g/d)	4.58	3.17	2.48	1.25	0.30	<0.001	<0.001	0.793
Absorbed (g/d)	5.62	5.92	2.65	3.74	0.25	<0.05	<0.001	0.231
ATTD of Ca (%)	55.3	66.8	52.3	75.1	2.8	<0.001	0.354	0.080

^1^Values are least square means.

ATTD: apparent total tract digestibility.

After 13 d of depletion, the daily P intake was reduced in the DON+ groups (*P* < 0.05; Table 3) and tended to be reduced in the Ca− groups (*P* = 0.068). The fecal P content was decreased by DON+ (*P* < 0.001) and Ca− (*P* < 0.05). The quantity of P absorbed tended to be lower for piglets receiving Ca− and DON− (DON × Ca; *P* = 0.059). The ATTD of P was increased by reducing the Ca level only in DON+ piglets (DON × Ca; *P* < 0.01). The daily Ca intake was decreased by Ca− (*P* < 0.001) and tended to be decreased by DON+ (*P* = 0.084). The fecal Ca was decreased by DON+ (*P* < 0.001) and Ca− (*P* < 0.001). The quantity of Ca absorbed was decreased by Ca− (*P* < 0.001) and increased by DON+ (*P* < 0.05). The ATTD of Ca tended to be more increased by DON+ in the Ca− (+ 44%) versus Ca+ (+ 21%) diets (DON × Ca; *P* = 0.080). In the serum sample, DON (*P* < 0.001) concentrations were increased in the DON+ groups (Table 4). There was no effect of DON contamination or dietary Ca on the plasma Ca, phosphate, and magnesium (Mg) concentrations after the 13-d depletion phase. The serum 25-OH-D_3_ concentration was decreased by DON+ (*P* < 0.05). The serum 1,25-OH_2_-D_3_ concentration was increased by Ca+ but only in DON+ diets (DON × Ca; *P* < 0.001).

**Table 4.** Impact of a Ca-deficient diet and deoxynivalenol (DON) contamination on blood parameters after the 13-d depletion phase^1^

Treatments	Ca+		Ca−		SEM	*P* value	Ca	DON × Ca
	DON−	DON+	DON−	DON+		DON		
DON (ng/mL)	2.89	19.1	1.00	19.1	2.03	<0.001	0.651	0.655
DOM-1 (ng/mL)^2^	0.24	4.53	-0.07	2.55	0.93	0.997	0.998	0.998
Calcium (mM)	3.29	3.16	3.24	3.25	0.04	0.316	0.702	0.224
Phosphate (mM)	3.86	3.78	3.89	3.97	0.08	0.988	0.344	0.456
Magnesium (mM)	0.74	0.71	0.73	0.75	0.01	0.804	0.334	0.274
25-OH-D_3_ (ng/mL)	10.2	8.41	10.3	8.15	0.59	<0.05	0.485	0.981
1,25-OH-D_3_ (pg/mL)^2^	169	154	165	211	15.0	<0.05	<0.001	<0.001

^1^Values are least square means.

^2^Analyzed with a Poisson adjustment.

DOM-1: deepoxy-deoxynivalenol; 25-OH-D_3_: 25-hydroxyl-Vitamin D_3_; 1,25-OH_2_-D_3_: calcitriol.

### Repletion phase

**Figure 1.**
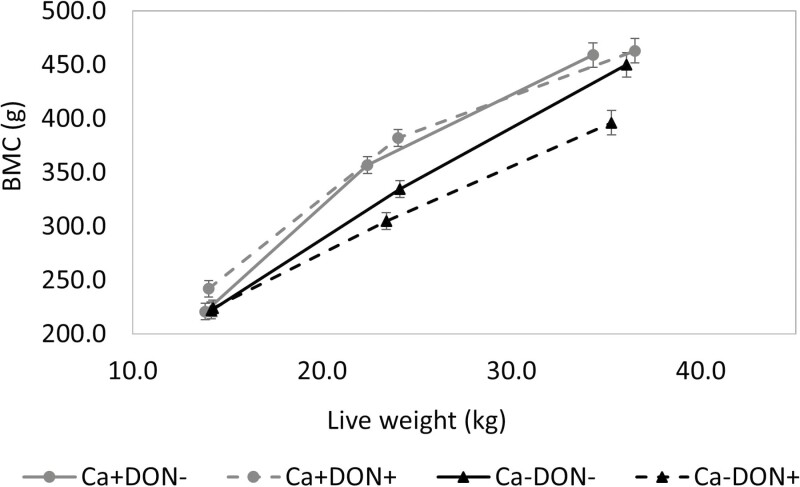
The effect of a Ca-deficient diet and exposure to deoxynivalenol (DON) contamination during a 13-d depletion phase on BMC relative to live weight and the recovery of bone mineralization under normal Ca level during the 14-d repletion phase. Data are reported as mean ± SEM.

**Figure 2.**
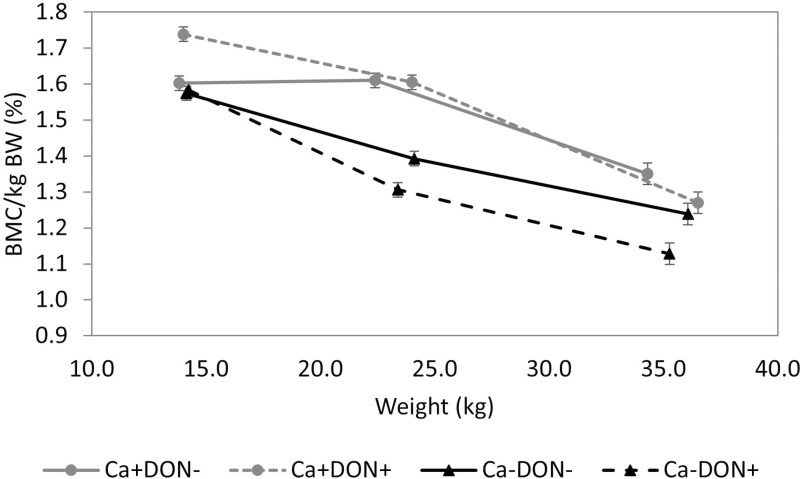
The effect of a Ca-deficient diet and the exposure to deoxynivalenol (DON) contamination during a 13-d depletion phase on BMC per body weight (%) relative to live weight and the recovery of bone mineralization under normal Ca level during the 14-d repletion phase. Data are reported as mean ± SEM.

After the 14-d repletion phase, all piglets received a Ca+ diet without DON contamination. The body weight of piglets previously receiving Ca− tended to be lower than Ca+ (*P* = 0.089; Table 5). The ADG was decreased by Ca− (*P* = 0.077) and increased by DON+ (*P* = 0.061). The ADFI also tended to decrease in Ca− piglets (*P* = 0.085), but the FCR was not modified by DON contamination or Ca level in the depletion phase. After the repletion phase, the BMC of piglets previously receiving Ca− tended to be lower when they had received DON+ (− 14%) versus DON− (− 1.5%; DON × Ca; *P* = 0.062; Figure 1). The BMC/kg of BW of piglets previously fed Ca− remained lower than Ca+ (*P* < 0.01; Figure 2). Piglets previously receiving Ca−/ DON+ had a reduced body P content (DON × Ca, *P* < 0.05) and tended to have a reduced body Ca content (DON × Ca, *P* = 0.060). During the repletion phase, the BMC (*P* = 0.083) and body Ca gains (*P* = 0.083) tended to be decreased by the previous DON contamination. The Ca efficiency (%) was increased by Ca− (*P* < 0.05) and tended to be decreased by DON+ (*P* = 0.081). The P efficiency (%) tended to be increased by Ca− (*P* = 0.064) and DON+ (*P* = 0.100).

**Table 5.** The effect of a previous exposure to Ca-deficient diet and deoxynivalenol (DON) contamination on growth performances and bone mineralization during the second 14-d repletion phase^1^

Actual treatment	Ca+		Ca+		SEM	*P* value	Ca	DON × Ca
Previous Treatments	Ca+		Ca−					
	DON−	DON+	DON−	DON+		DON		
Body weight (kg)	34.3	36.5	36.1	35.3	1.31	0.425	0.759	0.097
Body protein (g)	6261	6721	6534	6495	233	0.172	0.877	0.107
Body lipid (g)	4905	5013	5452	4858	346	0.427	0.521	0.254
BMC (g)	459	463	450	396	11.3	0.104	<0.05	0.062
BMC/BW (%)	1.35	1.27	1.24	1.13	0.05	0.050	0.012	0.738
Body Ca (g)	168	170	165	146	5.74	0.107	<0.05	0.060
Body P (g)	150	155	152	141	4.48	0.445	0.061	<0.05
Overall								
ADG (g/d)	956	991	894	959	21.2	0.061	0.077	0.552
ADFI (g/d)	1631	1673	1563	1573	48.2	0.579	0.085	0.731
FCR	1.71	1.69	1.75	1.65	0.05	0.186	0.897	0.416
BMC gain (g/d)	7.30	5.67	8.34	6.54	0.66	0.083	0.324	0.927
Body Ca gain (g/d)	2.70	2.12	3.07	2.42	0.24	0.083	0.326	0.921
Body P gain (g/d)	3.03	2.83	3.21	2.89	0.13	0.166	0.520	0.724
Ca efficiency^2^ (%)	25.1	18.5	28.0	26.4	2.25	0.081	<0.05	0.278
P efficiency^2^ (%)	26.2	23.0	27.5	26.4	1.23	0.100	0.064	0.401

^1^Values are least square means.

^2^Efficency was calculated by ratio between mineral retained estimated by DXA on mineral intake.

During the repletion phase, daily P and Ca intakes were not affected by Ca level and DON contamination. Fecal excretion of P was also not modified by previous dietary treatments. The daily P absorbed (DON × Ca; *P* < 0.05; Table 6) and the P ATTD (DON × Ca, *P* < 0.05) were reduced in piglets previously receiving the Ca+ DON− diet. Fecal Ca was decreased in the Ca− group (*P* < 0.05). The daily Ca absorbed and the Ca ATTD (DON × Ca; *P* < 0.05) were reduced in piglets previously receiving Ca+ without DON contamination. After the repletion phase, the plasma Ca concentration tended to be increased in the piglets in the Ca−/ DON− group (DON × Ca; *P* = 0.094), but the plasma P and Mg were not modified during the repletion phase (Table 7). The serum 25-OH-D_3_ was not modified during the repletion phase, but the serum 1,25-OH_2_-D_3_ was increased in the Ca− piglets, especially for the Ca−/ DON+ treatment (DON × Ca; *P* < 0.001).

**Table 6.** Digestible phosphorus and calcium in pigs measured during the second 14-d repletion phase^1^

Actual treatment	Ca+		Ca+		SEM	*P* value	Ca	DON × Ca
Previous treatments	Ca+		Ca−					
	DON−	DON+	DON−	DON+		DON		
Phosphorus								
Intake (g/d)	11.7	12.4	11.5	11.6	0.39	0.323	0.207	0.430
Feces (g/d)	6.49	5.84	5.29	5.58	0.48	0.790	0.284	0.487
Absorbed (g/d)	5.23	7.72	6.26	6.04	0.45	<0.05	0.511	<0.05
ATTD of P (%)	42.0	61.6	54.0	51.7	3.16	0.069	0.903	<0.05
Calcium								
Intake (g/d)	11.0	11.6	10.8	10.9	0.37	0.323	0.207	0.730
Feces (g/d)	4.73	4.77	3.42	3.59	0.44	0.868	<0.05	0.920
Absorbed (g/d)	6.26	8.03	7.40	7.31	0.33	<0.05	0.584	<0.05
ATTD of Ca (%)	57.5	68.1	68.5	66.9	2.18	0.145	0.114	<0.05

^1^Values are least square means.

**Table 7.** Impact of a previous Ca-deficient diet and deoxynivalenol (DON) contamination on blood parameters after the second 14-d repletion phase^1^

Actual treatment	Ca+		Ca+		SEM		Ca	DON × Ca
Treatments	Ca+		Ca−			*P* value		
	DON−	DON+	DON−	DON+		DON		
DON (ng/mL)	3.55	6.09	5.38	3.66	1.18	0.806	0.858	0.209
DOM-1 (ng/mL)^2^	0.00	0.25	1.05	-0.02	0.30	0.990	0.989	0.983
Calcium (mM)	3.12	3.20	3.31	3.15	0.05	0.566	0.366	0.094
Phosphate (mM)	3.64	3.57	3.77	3.73	0.11	0.714	0.323	0.923
Magnesium (mM)	0.64	0.66	0.68	0.67	0.01	0.654	0.156	0.489
25-OH-D_3_ (ng/mL)	7.35	9.60	9.76	8.62	0.94	0.547	0.870	0.259
1,25-OH-D3 (pg/mL)^2^	186	181	203	267	16.2	<0.01	<0.001	<0.001

^1^Value are least square means.

^2^Analyzed with a Poisson adjustment.

### Experiment 2

After 9 d of the trial, the ADG, ADFI, FCR, and BW were not affected by DON contamination (Table 8). After 5 d of the trial, the daily P intake and the fecal P were decreased in the DON+ group when compared with the DON− group (*P* < 0.05). The P ATTD was increased in piglets receiving DON+ (*P* < 0.01). The daily Ca intake (*P* < 0.01), fecal, and absorbed (*P* < 0.01) were decreased by DON+. The ATTD of Ca tended to be increased by DON+ compared with DON− (*P* = 0.062). DON contamination decreased plasma Mg (*P* < 0.01; Table 8) and tended to decrease plasma Ca (*P* = 0.062) concentrations. The Ca:P ratio was also decreased by DON+ compared with DON− (*P* < 0.01). The plasma P and serum 25-OH-D_3_ concentrations were not modified by DON contamination.

**Table 8.** The effect of a continuous exposure to deoxynivalenol (DON) contamination over 9 d on growth performance and blood parameters and over 6 d on digestible Ca and P^1^

Treatments	DON−	DON+	SEM	P-value
Growth performance				
ADG (g/d)	705	650	58.6	0.661
ADFI (g/d)	1235	1202	51.4	0.659
FCR	1.72	1.92	0.11	0.155
Body weight (kg)	21.1	21.0	0.62	0.868
Blood				
Ca (mM)	2.99	2.84	0.16	0.062
P (mM)	3.49	3.55	0.80	0.678
Mg (mM)	0.78	0.67	0.04	<0.01
Ca/P	0.88	0.80	0.03	<0.01
25-OH-D_3_ (ng/mL)	9.40	8.04	0.94	0.221
Digestibility				
Phosphorus				
Intake (g/d)	11.0	9.11	0.36	<0.05
Feces (g/d)	4.31	2.71	0.23	<0.05
Absorbed (g/d)	6.75	6.36	0.22	0.292
ATTD of P (%)	61.1	70.0	1.34	<0.01
Calcium				
Intake (g/d)	15.0	7.89	0.52	<0.01
Feces (g/d)	4.16	1.85	0.28	<0.01
Absorbed (g/d)	10.9	6.01	0.31	<0.01
ATTD of Ca (%)	72.4	76.3	1.12	0.062

^1^Values are least square means.

**Figure 3.**
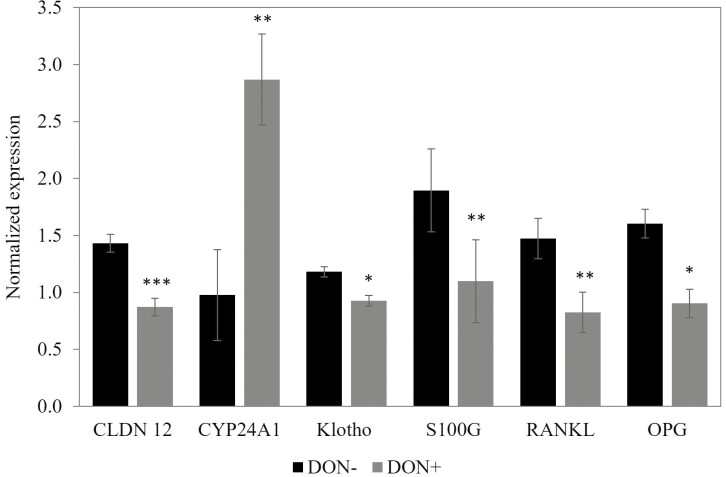
The effect of a continuous exposure to DON contamination over 10 d on gene expression in jejunum, kidney, and cortical femur tissues. CLDN12 and CYP24A1 were evaluated in jejunum tissue. Klotho and S100G were evaluated in kidney tissue. RANKL and OPG were evaluated in cortical femur tissues. CYP24A1 and S100G were analyzed with a Poisson adjustment. CLDN12: claudine 12; CYP24A1: 24-hydroxylase; Klotho: co-receptor to FGF23; S100G: Calbindin D9K; RANKL: receptor activator of NFκB ligand; OPG: osteprotegerin. Data are reported as mean ± SEM. *P < 0.10; **P < 0.05; ***P < 0.01.

After 9 d of DON contamination, there was no effect on liver expression of VDR and CYP2R1. In the jejunal mucosa, DON contamination increased CYP24A1 gene expression (*P* < 0.05; Figure 3) but decreased CLDN12 gene expression (*P* < 0.01). DON had no effect on the other genes evaluated in the jejunum (CALB-1, CLDN2, Klotho, S100G, SLC20A2, SLC34A3, TRPV6, VDR). In the kidney, DON contamination decreased S100G (*P* < 0.05) and tended to decrease Klotho (*P* = 0.075) gene expression. DON had no effect on the other genes evaluated in the kidney (CALB-1, SLC34A3, TRPV5, CYP27B1, SLC8A1, CYP24A1). In the cortical femur tissues, DON contamination decreased RANKL (*P* < 0.05) and tended to decrease OPG (*P* = 0.065) gene expression. DON had no effect on the other genes evaluated in the cortical femur tissues (CALCR, FGF23, Klotho, CYP27B1, VDR, OTC, Runx2) and no effect on the expression of these same genes was observed in the trabecular femur tissues.

## Discussion

The main objective of this study was to better understand the interaction between DON contamination and Ca and P metabolism. The contaminated feed had a lower DON concentration than expected, 2.7 instead of 3.5 mg/kg, the latter known to induce changes in growth performances (Wellington et al., 2020). We showed that a DON-contaminated diet (4.9 mg/kg) decreased piglet (6.4 kg) gene expression related to Ca and P intestinal and renal absorption, and induced an increase in bone mineralization relative to BW, suggesting that the timing chosen (9 d after the start of DON contamination) may be too late to assess the short-term effect of DON on gene expression related to Ca and P metabolism in intestinal and renal tissues. It is also possible that the gene expression related to these metabolisms is not correlated with the protein expression and functions in these tissues. Le Than et al. (2015) also observed an increase in Ca ATTD and retention efficiency and reduced Ca excretion in piglets (6 kg) fed a diet contaminated with approximately 4.0 mg/kg of DON, while no differences were observed for P (Le Thanh et al., 2015). With the aim of optimizing P utilization efficiency, previous research has used a depletion and repletion protocol (Lautrou et al., 2021), which activates Ca and P regulation (Létourneau-Montminy et al., 2014; Gonzalo et al., 2018). The effect of DON contamination in low and normal Ca diets, and also the ability of piglets to cope with the deficiency when dietary Ca is returned to normal levels was also studied. In the present study, the strategy used was a low dietary Ca, as proposed by Friggens et al. (2004), so that the intestinal absorption of Ca and P and the mobilization of Ca and P reserves in the body will be modulated according to the Ca and P availability in diet. During the depletion phase, the animal should be able to more efficiently absorb these minerals because of their hormonal regulation to maintain extracellular concentrations (Létourneau-Montminy et al., 2014).

### Impact of Ca deficiency and DON contamination during the depletion phase

During the depletion phase, the low dietary Ca decreased the feed intake, but it did not impact the ADG, leading to a decreased FCR. Previous studies have also reported similar findings for ADFI, but ADG was often reduced concomitantly. For instance, Lagos et al. (2019) observed that reducing Ca from 0.59% to 0.44% with normal P levels (0.50% or 0.42% STTD P) for 21 d in piglets (11 kg) reduced the growth and feed intake. Gonzalo et al. (2018) also observed that the ADG was decreased by a 28-d phase of low dietary Ca (0.95% vs. 0.65% Ca) in growing pigs (14 to 35 kg). However, some studies conducted on finishing pigs showed no effect of reduced Ca levels on growth performance (Bai et al., 2017; Merriman et al., 2017).

Several studies have reported decreased ADG and feed intake with DON contamination levels starting at 3.5 mg/kg (Andretta et al., 2012; Lessard et al., 2015; Wellington et al., 2020; Sauvé et al., 2023). However, in the present study, DON contamination did not modify growth performance, although piglets receiving DON+ showed improved FCR. Similar results have been observed in other studies involving DON-contaminated feed, with concentrations ranging from 0.84 to 3.1 mg/kg (Accensi et al., 2006; Le Thanh et al., 2016). The lack of effect on growth and feed intake in the present study may be because of the relatively short duration of DON exposure (9 and 13 d) and the lower DON dose (2.72 mg/kg). Indeed, the effect of DON on ADG and ADFI is dose and time dependent (House et al., 2002; Goyarts and Dänicke, 2005). Farming conditions may also influence the response to DON, as pigs can be exposed to lipopolysaccharide (**LPS**) which exacerbates the effect of DON on immune system stimulation (Kullik et al., 2013).

Regarding BMC, P and Ca retention efficiency during the depletion phase, an interaction between dietary Ca and DON was observed for many of the criteria measured. For instance, reducing dietary Ca resulted in decreased BMC at day 13 and a lower BMC gain. These reductions were more pronounced in the group exposed to DON+ than in the DON− group, with decreases of BMC and BMC gain of 20% and 43% for DON+ and 6% and 16% for DON−. Because Ca is mostly deposited in bone, a decrease in BMC gain during the depletion period can be expected, as previously observed, and reviewed by Lautrou et al. (2021). These reductions in BMC and BMC gain were less pronounced in DON− because the piglet can use various mechanisms to increase efficiency, or possibly because bone growth was less affected by the low Ca availability in the Ca−/ DON− group. This adaptation process following low Ca intake is generally regulated by the action of the PTH, which then leads to calcitriol synthesis (Brown, 2013). In conditions of low Ca levels, PTH secretion has been shown to increase renal Ca reabsorption (González-Vega and Stein, 2014), while calcitriol enhances Ca intestinal absorption (Christakos et al., 2014). Results of P and Ca digestibility after 5 d of depletion indicated higher ATTD of Ca (59 vs. 72%) and P (21 vs. 60%) when Ca levels were reduced in DON−, leading to higher absorption of P, although Ca absorption remained limited because of the restricted amount in the diet. González-Vega et al. (2016) also observed a decrease in Ca intake, Ca excretion, and in the quantity of Ca absorbed, which increased the Ca absorption (% intake) when growing pigs (13.2 kg) were fed a Ca-deficient diet (0.38% Ca) for 11 d (González-Vega et al., 2016). However, after 13 d, these differences were no longer present. In fact, Ca digestibility in the DON−/ Ca− group decreased from 72% to 52% from day 5 to day 13. Therefore, the adaptation to a Ca-deficient diet in pig is rapid but transitory. Previous experiments with the depletion–repletion protocol generally reduced both Ca and P levels (Lautrou et al., 2021), which limited the possibility of increasing the absorbed amount, as observed in this study for P.

Results showed the P and Ca efficiency in piglets responded differently to a decrease in dietary Ca when concomitantly facing a DON challenge. Indeed, compared with the piglets receiving the DON− diet, a reduction in dietary Ca resulted in a greater reduction in BMC and altered P and Ca efficiency in the DON+ diet. It is possible that the lower P content in the feed of the Ca− DON+ treatment compared with the other treatments also limited bone deposition—as did the lower Ca content—and increased the Ca and P bone resorption. The Ca and P retentions were reduced in Ca−/ DON+ despite higher or similar Ca and P absorbed at day 13. A modification in the Ca balance was also observed by Le Than et al. (2015), as piglets (6 kg) increased their Ca retention and digestibility and reduced Ca excretion when fed a diet contaminated with approximately 4 mg/kg of DON but containing approximately 1% Ca. This value is 25% higher than the level used in the Ca+ group but 3 times higher than the level in the Ca− diets. However, no differences were observed for P retention and absorption (Le Thanh et al., 2015). Therefore, the effect of DON on Ca and P retention appears to be dependent on the level of Ca in the diet. Previous research has observed that DON alters the intestinal barrier functions by increasing the membrane permeability, thereby enhancing both transcellular (through enterocytes) and paracellular (through tight junction proteins) nutrient transport, including Ca (Pinton et al., 2009, 2010; Payros et al., 2016). Hypocalcemia is also known to stimulate the Ca and P intestinal absorption through the inhibition of the CaSR, leading to increased PTH secretion (Karsdal et al., 2008; Wu et al., 2016). Therefore, the combination of low Ca levels and a DON-contaminated diet further enhanced the Ca and P ATTD at day 13 without affecting Ca efficiency, but reduced P efficiency. The fact that the Ca, P and BCM gain of piglets receiving Ca− DON+ was much lower than those receiving Ca− DON− suggests that a combination of low Ca and DON contamination altered the renal reabsorption of Ca and P or negatively affected the mineral accretion at the bone level during the depletion phase. The DON contamination period in this study was relatively short, and it remains uncertain whether pigs can adapt to long-term exposure, or if the bone mineralisation will continue to be impacted by alterations in phosphocalcic metabolic regulation. Under normal Ca levels, DON contamination increased BMC and BMC gain by 7% and 5%, respectively, consistent with our previous findings in piglets exposed to DON for 21 d (Sauvé et al., 2023), which is the result of the increase in Ca and P ATTD observed after 5 and 13 d.

In this study, serum 25-OH-D_3_ and 1,25-OH_2_-D_3_ concentrations were decreased by DON contamination, consistent with previous findings (Sergeev et al., 1990; Sauvé et al., 2023). However, in Ca-depleted piglets receiving DON-contaminated feed, serum 1,25-OH_2_-D_3_ was significantly increased. With lower Ca levels comes an increase in the production of calcitriol to increase intestinal Ca and P absorption (Christakos et al., 2014) and stimulate the formation and activity of osteoclasts, resulting in the release of Ca and P from bone (Goltzman, 2018). Therefore, this increased calcitriol concentration explains the reduced 25-OH-D_3_ concentration in the Ca-depleted piglets receiving DON-contaminated feed, as 25-OH-D_3_ is directly hydroxylated into calcitriol (Christakos et al., 2014). However, the serum concentrations of Ca, P, and Mg after the 13 d of depletion phase were not modified.

### Impact of Ca deficiency and DON contamination strategies during the repletion phase

During the second phase, known as the repletion phase, all pigs received the same diet with normal Ca levels (0.65%) and without DON-contaminated ingredients. The previous Ca deficiency tended to reduce the pig growth performance during the repletion phase. This might be attributed to the age of the pigs; previous observations have indicated that younger pigs are more negatively affected by Ca and P dietary deficiencies than older pigs (Letourneau-Montminy et al., 2015; Gonzalo et al., 2018) because they have lower mineral reserves and higher mineral requirements (NRC, 2012).

The response of piglets to the repletion phase still depended on the dietary Ca they received during the depletion phase, and this response differed if they faced a DON challenge. For instance, Ca and P absorbed (g/kg) and the ATTD of Ca and P were more reduced in Ca+ piglets, but only when there was no DON present. The Ca− pigs also had higher Ca and P efficiency, confirming our hypothesis regarding the effect of a depletion on Ca and P utilization. Other studies have also observed increased utilization efficiency of Ca or P following a depletion phase (Sommerville et al., 1985; González-Vega et al., 2016). Interestingly, 14 d after feeding DON-contaminated feed, the ADG was increased in piglets previously challenged with DON, showing the capacity of young pigs to recover from a 13-d continuous DON exposure. The quantity of Ca and P absorbed (g/kg) and the ATTD of Ca and P were increased in the DON+ group when they had previously received Ca+. However, this increase in ATTD did not result in improved BMC, Ca, and P gains during the repletion phase for Ca− pigs.

While BMC gain was not significantly affected by the Ca− diets, piglets receiving Ca− without DON were able to recover their deficit in BMC after the repletion phase, reaching levels comparable with the Ca+ group, although their BMC/kg of BW was still lower. Piglets receiving a Ca− diet also had increased Ca and P efficiency (%) and a tendency to have elevated plasma Ca concentrations. Piglets that received the Ca− DON− diet had similar body Ca and P regulation compared with the piglets receiving Ca+ DON−. Bone mineralization is a slow process and varies depending on the duration of the depletion phase and the age of the animals. Younger pigs tend to deplete their bone minerals more rapidly and extensively than older pigs (Létourneau-Montminy et al., 2014). In a previous study, finishing pigs weighing approximately 60 kg were able to recover their bone mineralization deficit after a 30-d repletion phase (0.79% Ca) that was preceded by a 30-d Ca deficiency (0.34%) period (Bai et al., 2017). Aiyangar et al. (2010), in contrast, observed that after 6 wk of a high-Ca diet (150% of established Ca requirements), piglets at 39 d of age still had lower whole-body bone mineralization following a 4-wk period of Ca deficiency (70% of established Ca requirements), although they achieved a similar gain in BMC as the control piglets (Aiyangar et al., 2010). However, piglets previously exposed to DON+ were not able to recover the BMC deficit caused by the Ca-depletion; their body Ca and P remained lower. Previous DON contamination also tended to decrease the Ca and P efficiency (%) despite an increase in intestinal Ca and P absorption. Serum 1,25-OH_2_-D_3_ concentration was also still increased in Ca− DON+ piglets. This suggests that the PTH production of those piglets might have been elevated, but the levels were too low in plasma EDTA to be evaluated. A rapid increase of calcitriol in serum can often mean an insufficient production of the calcitriol itself, as it is usually well feedback regulated (Schmidt-Gayk et al., 1997). The Ca− DON+ piglets might still have increased bone resorption, releasing Ca from the bone through the activation of osteoclasts and inhibition of osteoblasts, along with increased intestinal Ca absorption (González-Vega and Stein, 2014), as evidenced by their numerically lower BMC and body Ca gains compared with the other groups.

### Impact of DON contamination on gene expression and blood parameters of euthanized piglets

Considering the increased Ca and P digestibility in piglets exposed to DON described in the first experiment, 16 piglets receiving either a DON+ or DON − diet with normal Ca levels were euthanized after 9 d of the trial to evaluate changes in gene expression and blood parameters related to Ca and P intestinal absorption and renal reabsorption and bone remodeling. Multiple genes were assessed in the jejunum, kidney, and femur cortical or trabecular tissues, but only significant results will be discussed. The Ca and P balance was also evaluated after 5 d of trial. It should be noted that the DON contamination did not impact the growth performance of these piglets.

There was no effect of DON contamination observed in trabecular femur tissue, possibly related to the high turnover rate of trabecular bone (Karsdal et al., 2008). In the liver, no significant effect of DON contamination was observed on genes related to vitamin D. In the jejunal mucosae, the CYP24A1 gene expression, responsible for the degradation of calcitriol into 24,25-OH_2_-D_3_ (Christakos et al., 2010), was upregulated by 78% in DON-contaminated piglets. The increased calcitriol catabolism into the inactive form 24,25-OH_2_-D_3_ partly explains the lower concentration of 25-OH-D_3_ observed in this study for DON+ after 13 d, as well as in our previous study (Sauvé et al., 2023); however, after 9 d, the 25-OH-D_3_ concentration was only numerically lower for DON+, but this was not significant. In the intestine, the 24-hydroxylase activity was generally enhanced by calcitriol and calcitonin (Tryfonidou et al., 2003).

Interestingly, the Ca concentration and ratio of Ca:P in plasma decreased with the continuous exposure to DON over 9 d. The Ca intake (− 47%) and excretion (− 56%) and the quantity of Ca absorbed (− 45%) were decreased by DON contamination, and the ATTD Ca (+ 5.4%) tended to increase. The lower Ca quantity absorbed is probably the cause of the reduced blood Ca concentration after 9 d, leading to a reduction in Ca excretion. The DON-contaminated diet also decreased plasma Mg concentration, which is closely linked to Ca and P metabolism. Hypomagnesemia is typically caused by gastrointestinal and renal losses because the extracellular Mg concentration is tightly regulated by the gut and kidneys (Tseng et al., 2022). DON contamination is known to increase apoptosis of cells, with the main target being high protein turnover tissues like the epithelial cell wall of the intestine. This disruption of the intestinal barrier can lead to an increased permeability and local intestinal inflammation (Payros et al., 2016; Ghosh et al., 2020). The barrier permeability mechanism has often been described in previous studies, although it was not specifically evaluated in this study.

However, the CLDN12 gene expression, encoding a tight junction protein involved in the paracellular transport of Ca (Fujita et al., 2008), was downregulated by 39% in response to the DON challenge. This suggests a negative regulation of the tight junction protein CLDN12 by DON, although this could imply a protein level either high or low. In IPEC-1 porcine cells, it was observed that tight junction protein CLDN4 expression was downregulated by DON, while its gene expression was upregulated (Payros et al., 2016). During hypocalcemia, transcellular transport becomes more active to enhance Ca intestinal absorption through the gut barrier, primarily controlled by calcitriol (Brown, 2013; González-Vega and Stein, 2014). However, DON contamination did not impact the transient receptor potential vanilloid channel TRPV6, responsible for the intestinal apical influx of active Ca transport and regulated by 1,25-OH_2_-D_3_ concentration (van de Graaf et al., 2004). In the kidney, the gene expression of S100G, involved in Ca transport (Pu et al., 2016), was decreased by 42% in piglets fed the DON-contaminated diet, which may have altered Ca reabsorption. Therefore, the reduction of plasma Ca levels by DON might have been caused by negative regulations of both paracellular intestinal transport and renal transport of Ca in response to the reduced Ca intake and quantity of Ca absorbed in the first 9 d.

The gene expression of Klotho, which acts as the co-receptor for FGF23, was reduced by 22% in response to the DON-contaminated diet. The Klotho gene is regulated, in part, by calcitriol and modulates renal P reabsorption (Bian et al., 2014). The FGF23 is a hypophosphatemic hormone that increases renal excretion of P, modulates the bioactivation of calcitriol (Haussler et al., 2012) and inhibits PTH production (Clinkenbeard and White, 2016). The reduction in Klotho expression may be explained by the decrease in P intake (−17%) and P excretion in feces (−37%) observed after 5 d of trials. A reduction in P excretion was also observed in piglets from 9 to 14 d after beginning the consumption of feed contaminated with 4 mg/kg of DON (Le Thanh et al., 2015).

The expression of the RANKL and OPG genes in the cortical femur was also decreased by 44% in animals fed DON-contaminated feed. The RANKL, through its interaction with the RANK receptor, induces the production and activation of osteoclasts responsible for bone resorption. In contrast, OPG acts as a receptor that binds to the same ligand as the RANK receptor, RANKL, but exerts an antagonistic effect by inhibiting osteoclast production. As a result, the OPG–RANKL complex counterbalances the bone resorption process initiated by the RANK–RANKL complex (Kohli and Kohli, 2011). Therefore, it is possible that DON contamination—by modifying the regulation of PTH, calcitonin, and calcitriol—led to a decrease in RANKL expression and consequently a reduction in the bone resorption process (O’Brien, 2010). This could explain the increased BMC in DON+ piglets, which was also observed in our previous experiment (Sauvé et al., 2023), and may provide insight into the lack of recovery in bone mineralization observed in DON+ piglets in this study.

## Conclusion

Short-term exposure to DON-contaminated feed resulted in modifications to the bone deposition process, depending on the Ca levels. At low Ca levels, BMC gain was highly reduced, yet Ca and P efficiency was not increased. The DON contamination increased intestinal absorption of Ca and P after 5 and 13 d, concomitant with previous studies that reported an increase in permeability of the intestinal barrier membrane caused by DON exposure, but this will need further confirmations. Piglets receiving DON presented reduced serum 25-OH-D_3_ levels to increase their 1,25-OH_2_-D_3_ serum levels when fed a Ca-deficient diet. As expected, the Ca deficiency induced regulations in phosphocalcic metabolism, increasing the Ca bone retention efficiency while decreasing bone mineralization and body Ca. However, Ca deficiency increased the intestinal absorption of Ca and P after 6 d. Following the repletion phase, the previous Ca deficiency enhanced the Ca and P utilization efficiency and intestinal digestibility, allowing pigs to recover from the bone mineralization deficit, despite reduced growth performances. Conversely, piglets exposed to DON and receiving the Ca-deficient diet did not recover from the bone mineralization deficit. In fact, their body Ca and P remained low, leading to elevated calcitriol concentrations and enhanced Ca and P intestinal absorption. After a continuous short-term exposure to DON over 9 d with normal Ca levels, pigs showed decreased expression of genes involved in intestinal and renal Ca transport, P excretion, and osteoclast production, which might have contributed to the reduced plasma Ca concentration. This reduction in circulating Ca by DON was also associated with a decreased Ca intake and quantity of Ca absorbed, although the P and Ca digestibility increased.

## Supplementary Material

skae099_suppl_Supplementary_Appendix

## Data Availability

The data sets generated during the current study are available from the corresponding author upon request.

## Abbreviations

1,25-OH_2_-D_3_: calcitriol
ADFI: average daily feed intake
ADG: average daily gain
ATTD: apparent total tract digestibility
BMC: bone mineral content
BW: bodyweight
CALB-1: calbindin D28K
Ca: calcium
CaSR: calcium-sensing receptor
CLDN: claudin
CYP24A1: 24-hydroxylase
CYP27B1: 1α-hydroxylase
CYP2R1: 25-hydroxylase
DON: deoxynivalenol
DXA: dual-energy X-ray absorptiometry
FGF23: fibroblast growth factor
OPG: osteoprotegerin
OTC: osteocalcin
P: phosphorus
PTH: parathormone
RANKL: factor receptor activator of nuclear factor-κB ligand
S100G: calbindin D9K
SLC20A2: Na-Pi type III transporter
SLC34A3: Na-Pi type IIc, renal phosphate co-transporter
SLC8A1: Na^2^+/Ca^2^ + 1 exchanger
qPCR: quantitative polymerase chain reaction
TRPV5: transient receptor potential vanilloid 5
TRPV6: transient receptor potential vanilloid 6
VDR: vitamin D receptor.
