# Supplementary material for: Impact of deoxynivalenol in a calcium depletion and repletion nutritional strategy in piglets
Source: J Anim Sci. 2024 Apr 13;102:skae099. doi: 10.1093/jas/skae099 (PMC11056887; doi:10.1093/jas/skae099)
Supplement: skae099_suppl_Supplementary_Appendix [file skae099_suppl_supplementary_appendix.docx]

**Appendix**

Table A1: Primer sequences used for qPCR

| Gene | Primer sequence (5'-3') | Product size, bp | Genebank accession no |
| --- | --- | --- | --- |
| *Vitamin D* |  |  |  |
| CYP2R1 | (F) TTCATCCCTTCTTGACTCCAAC | 201 | XM_003480731 |
|  | (R) TTTATCTGTCACCTGTCACCAC |  |  |
| VDR | (F) AGGCTTCTTCAGACGGAGCAT | 143 | NM_001097414.1 |
|  | (R) ACTCCTTCATCATGCCGATGT |  |  |
| CYP27B1 | (F) TGGGCTCTCTATGAACTCTCTC | 157 | DQ295065.1 |
|  | (R) GTCTTAGCACTTCCTTGACCAC |  |  |
| CYP24A1 | (F) TGTGACGAGAGAGGCTGCATTGAA | 177 | AF245504.2 |
|  | (R) TCATCTTCCCGAACGTGCTCATCA |  |  |
|  |  |  |  |
| *Phosphorus* |  |  |  |
| Klotho | (F) ACGCGGAACATGACGTACAG | 121 | XM_013989399.1 |
|  | (R) CCTGCAAGGCGATGGAGAT |  |  |
| SLC20A2 | (F) GTGCACCTGCTCTTCCACTTC | 106 | XM_005657658.1 |
|  | (R) ACAAAGCTACCAGAGGACCAATG |  |  |
| SLC34A3 | (F) CTACCTGTTGCTCGGCTTC | 117 | XM_021081180.1 |
|  | (R) ACGATGAGGACAATCAGCAC |  |  |
|  |  |  |  |
| *Calcium* |  |  |  |
| S100G | (F) GAAGGAGGAGCTGAAGCAACTG | 139 | NM_214140.2 |
|  | (R) CACTAACACCTGGAATTCTTCAAAAC |  |  |
| CALB-1 | (F) TGGATCAGTATGGGCAAAGAGA | 133 | NM_001130226.1 |
|  | (R) GTCTTCATGAATTCCTCACAGGACTT |  |  |
| TRPV5 | (F) GCTGCGAGTACGTCGCTATGT | 86 | XM_003484001.2 |
|  | (R) CAGAGGGCTGTTTCTCAGAGAGA |  |  |
| SLC8A1 | (F) AATTGCTAGAGCTACTGTGTATTTCG | 136 | FJ268730.1 |
|  | (R) ATTGGGCTTCTTTATGGTTATTTCT |  |  |
| TRPV6 | (F) TGGGTGTCCCAAAGTCCAAG | 95 | XM_013985575.1 |
|  | (R) ACTCCCTCCTCCTCCCAAAT |  |  |
|  |  |  |  |
| *Cortical and trabecular femur* |  |  |  |
| OPG | (F) AAGTCCCGACAGAAGACATCGAGGA | 148 | XM_003481346.4 |
|  | (R) GGTACGTCTTGAGATGCTTCAGGGC |  |  |
| RANKL | (F) ACCTGTATGCCAACATCTGCTTTCG | 227 | DQ523616.1 |
|  | (R) TGCTGATTTCCTCGCCAGATCGT |  |  |
| OTC | (F) TACCCAGATCCTCTGGAGCCC | 110 | AY150038.1 |
|  | (R) TATGCCATAGAAGCGCCGATA |  |  |
| Runx2 | (F) CAAGTGCGGTGCAAACTTTCTCCA | 185 | XM_003482203.4 |
|  | (R) AGGCTGTTTGATGCCATAGTCCCT |  |  |
| *Reference* |  |  |  |
| GAPDH | (F) CCC CAA CGT GTC GGT TGT | 91 | XM_021091114.1 |
|  | (R) CTC GGA CGC CTG CTT CAC |  |  |
| β-Actin | (F) CAT CAC CAT CGG CAA CGA | 128 | XM_003357928.4 |
|  | (R) GGA TGT CGA CGT CGC ACTT |  |  |
| HPRT | (F) TTG TGG TAG GCT ATG CCC TTG ACT | 117 | NM_001032376 |
|  | (R) CTC AAC TTG AAC TCT CCT CTT AGG |  |  |
